# High-rising epiglottis, an uncommon cause of dysphagia

**DOI:** 10.11604/pamj.2021.39.247.30459

**Published:** 2021-08-18

**Authors:** Husam Bader, Alexandra Rubin

**Affiliations:** 1 Monmouth Medical Center, New jersey, United States of America,; 2 Rutgers Robert Wood Johnson Medical School, New Jersey, United States of America

**Keywords:** Dysphagia, high-rising epiglottis, internal medicine

## Image in medicine

A 43-year-old female, 147cm in height, with a medical history significant for sickle cell disease, chronic pain with chronic opioid dependence and diastolic congestive heart failure. Patient was hospitalized for decompensated congestive heart failure and new onset of anasarca. Additionally, the patient described a sensation of “fullness” in her throat for over a decade, but denied other gastrointestinal symptoms including weight loss, choking, nausea or vomiting. Physical examination revealed a “high-rising epiglottis”. There was no history of epiglottitis. Further work-up of the dysphagia was otherwise unremarkable. A high-rising epiglottis is a benign entity that is rarely described in adults, particularly those with short stature and can result in dysphagia.

**Figure 1:**
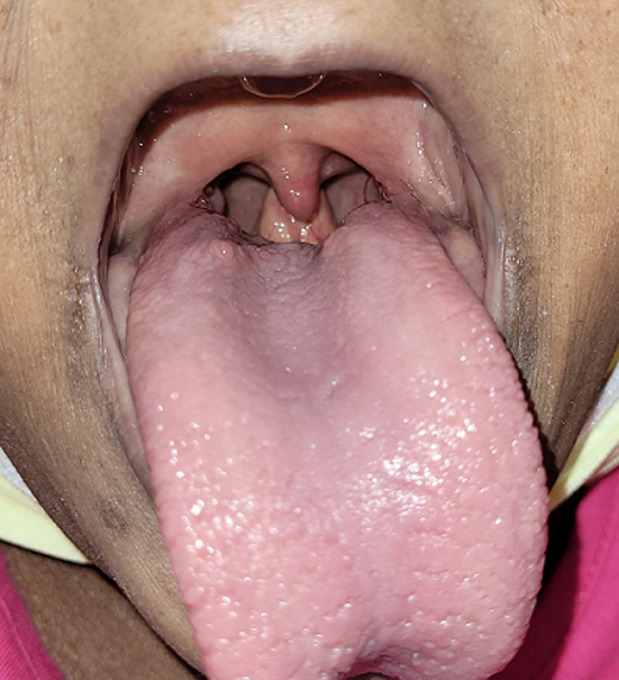
high-rising epiglottis

